# Depolarization versus repolarization abnormality underlying inferolateral J-wave syndromes: New concepts in sudden cardiac death with apparently normal hearts

**DOI:** 10.1016/j.hrthm.2018.10.040

**Published:** 2019-05

**Authors:** Michel Haïssaguerre, Koonlawee Nademanee, Mélèze Hocini, Ghassen Cheniti, Josselin Duchateau, Antonio Frontera, Frédéric Sacher, Nicolas Derval, Arnaud Denis, Thomas Pambrun, Rémi Dubois, Pierre Jaïs, David Benoist, Richard D. Walton, Akihiko Nogami, Ruben Coronel, Mark Potse, Olivier Bernus

**Affiliations:** ∗ Bordeaux University Hospital, Bordeaux, France; † IHU LIRYC, Electrophysiology and Heart Modeling Institute, Bordeaux, France; ‡ University of Bordeaux, U1045, Bordeaux, France; § Bumrungrad Hospital, Bangkok, Thailand; ‖ University of Tsukuba, Ibaraki, Japan

**Keywords:** Early repolarization, J-wave syndrome, Sudden death, Ventricular fibrillation

## Abstract

Early repolarization indicates a distinct electrocardiographic phenotype affecting the junction between the QRS complex and the ST segment in inferolateral leads (inferolateral J-wave syndromes). It has been considered a benign electrocardiographic variant for decades, but recent clinical studies have demonstrated its arrhythmogenicity in a small subset, supported by experimental studies showing transmural dispersion of repolarization. Here we review the current knowledge and the issues of risk stratification that limit clinical management. In addition, we report on new mapping data of patients refractory to pharmacologic treatment using high-density electrogram mapping at the time of inscription of J wave. These data demonstrate that distinct substrates, delayed depolarization, and abnormal early repolarization underlie inferolateral J-wave syndromes, with significant implications. Finally, based on these data, we propose a new simplified mechanistic classification of sudden cardiac deaths without apparent structural heart disease.

## Graphical abstract

**Figure:**
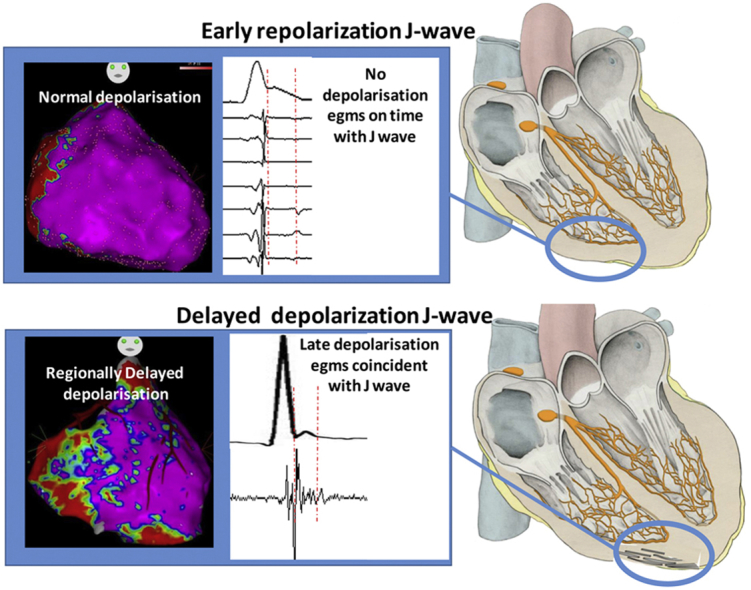

Early repolarization indicates a distinct electrocardiographic (ECG) phenotype affecting the junction (J point or J wave) between the QRS complex and the ST segment in inferolateral leads. It was initially described as a benign ECG finding or found in association with hypothermia.^1^, ^2^, ^3^, ^4^ Subsequently, many other conditions producing this phenotype have been described, such as hypercalcemia, acute ischemia, and brain injury.

The link with an increased risk of arrhythmic death was later demonstrated in sporadic cases,^5^, ^6^ in case control studies of unexplained sudden cardiac death (SCD), and in association with various types of structural heart disease (SHD).^7^, ^8^, ^9^, ^10^, ^11^ This article focuses on J-wave syndromes affecting inferolateral leads and reviews the current knowledge and the limitations in risk stratification. In addition, we report new clinical mapping data using high-density electrode mapping, which provides evidence that 2 distinct substrates, delayed depolarization and early repolarization abnormality, underlie inferolateral J-wave syndromes.

## Diagnosis of early repolarization/inferolateral J-wave syndrome

Expert consensus recommendations^12^, ^13^ distinguish an early repolarization ECG pattern (ER) and an early repolarization syndrome. The pattern is defined as the presence of J-point elevation ≥1 mm in ≥2 contiguous inferior and/or lateral leads of a standard 12-lead ECG. The syndrome is said to be present when the pattern is found in (1) a patient resuscitated from otherwise unexplained ventricular fibrillation (VF); or (2) an SCD victim with a negative autopsy and medical chart review with a previous ECG. The J-point elevation may be a notch or a slur, with or without elevation of the ST segment.

**Figure 1:**
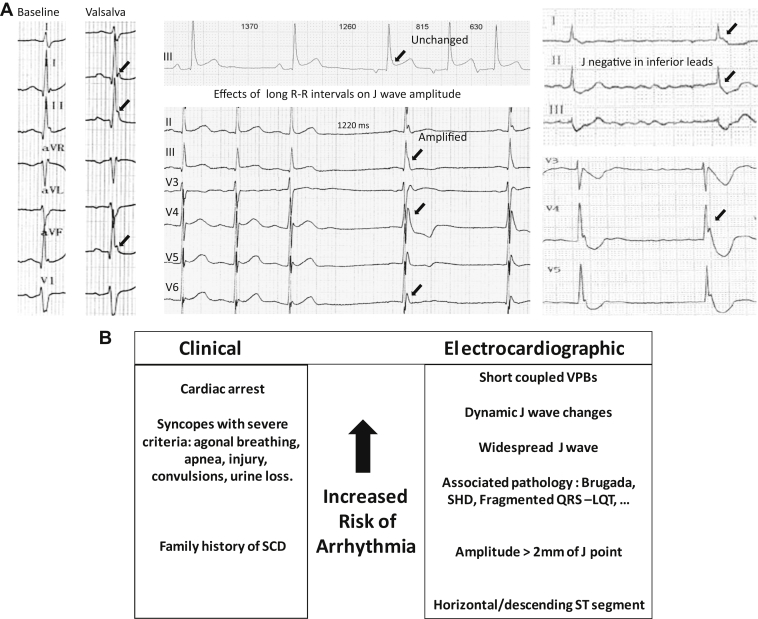
**A:** Electrocardiographic (ECG) variations in inferolateral J waves. **Left:** Valsalva or strong inspiration maneuver producing J-wave amplification. **Middle:** Cycle length prolongation associated with either unchanged pattern or amplification. **Right:** Negative J waves in inferior leads and positive J waves in lateral leads. Note the increase of J wave after the pause (arrow). **B:** Hierarchical view of ECG and clinical risk factors. LQT = long QT; SCD = sudden cardiac death; SHD = structural heart disease; VPB = ventricular premature beat.

The definition of J wave is sometimes ambiguous because of the small amplitude and spontaneous changes of the signal. J/ST elevation in inferior leads may be more easily missed as the pattern is less stereotypical than the Brugada syndrome (BrS). Bipolar limb leads (I, III, aVF) may be less sensitive than unipolar precordial leads in identifying discrete J/ST-segment abnormalities and in assessing the spatial extent of the J wave. Although no emphasized previously, a J wave may also be negative and less apparent when it follows a negative QRS complex. Figure 1A illustrates these ECG variations.

## Clinical significance

The ECG pattern of early repolarization was first reported in 1936 as a normal variant.^1^ In 1938, Tomaszewski^2^ described a slow deflection between the QRS complex and the ST segment in an accidentally hypothermic man. In 1953, Osborn^3^ described a “current of injury” in experimental acidosis and hypothermia in dogs and which was often associated with the development of VF.

The ER was known for decades to be an innocent ECG feature that was more common in young men and in athletes, with a prevalence varying between 1% to 24 % in the general population. However, case reports of unexpected SCD associated with an inferolateral J-wave abnormality were published as early as 1984, most of them concerning victims from Japan and Southeast Asia.^5^, ^6^, ^7^, ^8^ Then, the potential arrhythmogenicity of ER was shown in wedge preparations by Yan and Antzelevitch.^14^ Finally, a definitive association between J waves and the pathogenesis of idiopathic VF was described in case control studies.^7^, ^8^, ^9^, ^10^, ^11^, ^15^, ^16^, ^17^, ^18^, ^19^ Although these results have raised some concerns for the affected patients, the high prevalence of J waves in the general population causes them to portend a low absolute arrhythmia risk.^20^, ^21^

### Risk in asymptomatic subjects

The prevalence of J waves in athletes is reported to be as high as 44%.^22^, ^23^, ^24^, ^25^ In a study of 704 athletes (14% with a J wave) with follow-up of 6 years, there was no observation of arrhythmic events.^24^ Similarly, no association with higher risk of death was observed during long-term follow-up in young adults with J waves.^25^ Interestingly, a correlation was found between J-point elevation and septal hypertrophy, as well as a possible link with exercise-induced hypertrophy.^23^, ^25^, ^26^, ^27^ False tendons have also been related to the genesis of J waves, potentially through altered depolarization in the Purkinje system due to localized stretching.^28^ Although J waves are common in African Americans, it is estimated that there is no increased risk in this subgroup, whereas a higher risk may be present in Asian populations.^29^, ^30^, ^31^ Noteworthy, the definition of ER in the previous literature varies in terms of J-wave or ST-elevation pattern; it may include individuals with only ST elevation and no J wave, which impacts on the reported prognostic value.

### Risk in association with SHD

Many studies have suggested that J waves associated with SHD increase the vulnerability to ventricular arrhythmias but not to nonarrhythmic cardiac events.^32^, ^33^, ^34^, ^35^, ^36^, ^37^ In a meta-analysis of 19 studies including 7268 SHD patients, a higher risk was associated with J waves specifically in inferior leads, with a notching configuration, and a horizontal or descending ST segment.^11^ An increased arrhythmic risk has also been shown in the event of myocardial ischemia.^32^ In an animal model, J waves appearing during left anterior descending artery occlusion were associated with 53% risk of VF.^34^ J waves, particularly in the inferior leads, were also associated with increased risk in patients with chronic coronary artery disease, or with dilated or hypertrophic cardiomyopathy.^35^, ^36^, ^37^ Therefore, the presence of inferior J waves associated with SHD increases the incidence of arrhythmic events. J waves and their association with other ECG abnormalities (eg, Brugada, long QT, and short QT syndromes) also increase the risk of arrhythmias.^38^, ^39^, ^40^, ^41^

### J waves and idiopathic VF: Risk stratification

Among patients with a history of idiopathic VF, several reports have provided clinical evidence of an increased prevalence of J-wave patterns. In the most recent study from Korea, J waves were observed in 35 of 81 patients (43%) with idiopathic VF and were associated with a higher risk of recurrences.^19^ The incidence of idiopathic VF associated with inferolateral J waves is estimated to be 3:100,000. This represents a low absolute risk compared with a prevalence of 1% to 24 % in the population.^7^, ^9^, ^15^, ^16^, ^17^, ^18^, ^19^ Therefore, the majority of individuals with ER are at minimal risk for arrhythmic events, so asymptomatic patients with no family history of SCD should be reassured.^20^, ^21^

In symptomatic patients, several ECG markers have been assessed in relation to outcome. However, the spontaneous variability of the J-ST pattern is a limiting factor. Clinical variables such as gender or ethnicity have provided conflicting results in terms of prognostic value. A family history of sudden death has been reported to be more common in association with SCD^42^ and likely increases the individual risk. The prognostic value of QRS slurring vs notching, often coexisting or changing in time, is still unclear. The following variables have been shown to be associated with an increased risk of malignant arrhythmias: (1) a horizontal or descending type of ST segment in the inferior leads, as opposed to an upward ST segment^15^, ^16^, ^17^, ^25^, ^31^; however the prevalence of this more risky ST pattern in controls may be up to 3%, reducing its specific value; (2) a higher magnitude of J waves (>2.0 mm) in the inferior leads^9^; and (3) an extensive ECG pattern involving anterior (leads V_1_–V_3_) and inferior leads.^7^, ^18^, ^41^

A consistent observation at the time of malignant arrhythmias is amplification of inferolateral J waves, which then recedes after spontaneous or pharmacologic arrhythmia termination.^6^, ^7^, ^43^, ^44^ A higher J-wave amplitude recorded soon after a syncope (compared with previous or subsequent ECGs) suggest that a malignant arrhythmia may have occurred. The variability of J waves after longer cycle lengths (post pause) is, in our opinion, an essential marker of electrical vulnerability. Unfortunately, there are no specific means to challenge the dynamicity of J-wave patterns in vulnerable patients (eg, like sodium blocker provocation in BrS). A Valsalva maneuver or Holter monitoring are useful to determine the J-wave dynamics on 12-lead ECG during cycle length variations. Finally, VF induction maneuvers during an electrophysiological study are of little utility. In a multicenter study of 81 patients followed up by implantable cardioverter–defibrillator interrogations, VF inducibility could not predict the incidence of subsequent arrhythmias.^45^

In summary, based on different published reviews it seems that no clinically strong risk stratification can be performed to identify the small subset of patients at high risk and facilitate primary prevention. Genetic variant analysis or specific pharmacologic testing hopefully will become of prognostic importance in the future.^13^ Currently the decision for therapeutic prevention in high-risk patients (by implantable cardioverter–defibrillator or sometimes by quinidine) or loop recorder implantation in intermediate-risk patients is based on the severity of clinical variables and ECG patterns: T-wave negativity, J-wave amplitude, spatial extent of the J-wave pattern, and dynamic changes in the J wave.^12^, ^20^, ^21^ The presence of short-coupled premature beats is not a risk factor as it indicates imminent threat of VF. Figure 1B summarizes our hierarchical view of risk factors.

### Evidence for repolarization and depolarization abnormalities as distinct mechanisms

There is ongoing controversy on whether the “J-wave syndromes” (Brugada and inferolateral J waves) are due to repolarization or depolarization abnormalities. The repolarization mechanism is founded on studies in right or left ventricular wedge preparations demonstrating that J waves can be a consequence of a transmural repolarization gradient due to a differential distribution and function of the transient outward current.^8^ The depolarization mechanism is founded on studies demonstrating that structural discontinuities can cause conduction disturbances by current-to-load mismatch and display the phenotype of J waves.^46^ In humans, however, the current data indicate heterogeneous mechanisms.

### Brugada syndrome

In BrS, the best evidence in support of a depolarization abnormality in humans came from Nademanee et al^47^, ^48^ and other groups^49^, ^50^, ^51^, ^52^, ^53^ showing late fractionated electrograms on the epicardial side of the right ventricular outflow tract. These electrograms were then correlated with the presence of interstitial fibrosis and reduced gap junction expression in biopsies or autopsy studies from affected patients.^46^, ^48^, ^49^ Such microstructural abnormalities were also reported in previous anatomic studies by Martini et al^54^ and Corrado et al.^55^ Noteworthy, Antzelevitch's group has demonstrated that fractionated electrograms in animal wedge preparations can also be caused by repolarization disparities and concealed phase 2 reentry.^56^ Our current clinical experience with high-resolution epi–endocardial mapping techniques (at the full organ level) demonstrates that late epicardial fragmented potentials are continuous to the main depolarization front. This provides a strong argument in favor of a depolarization abnormality as a primary substrate of BrS in humans.

### Inferolateral J-wave syndrome

In inferolateral J waves, repolarization abnormalities have been well established as the dominant substrate. However, a slurred end of the QRS complex is also a well-known marker of delayed activation in SHD (termed peri-infarction block or epsilon wave).^57^ The J wave here indicates “postexcited” myocardium in the same way as the delta wave in Wolff-Parkinson-White syndrome indicates “preexcited” myocardium.

High-density invasive mapping data have been collected from 38 patients with inferolateral J-wave syndrome at 3 centers.^58^ These patients had no demonstrated SHD, and most were referred for VF recurrence despite treatment with antiarrhythmic drugs, including quinidine. Electroanatomic mapping was performed during sinus rhythm to obtain endocardial and epicardial electrograms (2000–6000 recorded points) in unipolar and bipolar mode (unipolar filters 0.05–250/500 Hz; bipolar filters 30–250/500 Hz). A 2-mm interelectrode spacing was used to minimize the recording of far-field potentials, and specific attention was paid to the electrograms coincident with the J wave. Abnormal electrograms were defined as prolonged fragmented electrograms with >3 components and a local duration >70 ms.^59^ These criteria are similar to those defining structural alteration and fibrotic tissue in SHD. The electrograms occurring within (and possibly prolonging) the J wave were considered as belonging to depolarization if they were sharp *and* in temporal and spatial continuity with the depolarization field mapped at the end of the QRS complex . They were considered as indicating ventricular repolarization if they either were not in continuity with the surrounding depolarization (presence of an electrical gap >100 ms) or displayed a slow pattern (hump) in unipolar mode that was not related to a near-field potential. Such slow potentials have been reported previously during direct or indirect epicardial recordings, in patients with J-wave or hypothermia conditions.^33^, ^60^, ^61^, ^62^, ^63^

Based on these definitions, the results indicate that the ECG J wave can be the phenotypic expression of either delayed depolarization or early repolarization, in the inferior part of ventricles.^58^ In patients who had concomitant BrS (spontaneous or provoked by a sodium channel blocker), inferolateral J waves were consistently caused by delayed depolarization of inferior myocardium. In patients without BrS, inferolateral J waves were caused by delayed depolarization in 24% of patients, whereas an ER was the cause of J waves in 76%. Note that the true prevalence of depolarization vs repolarization abnormalities may be different, as the patients were often only referred after quinidine failure, which likely favors a higher proportion of patients with delayed repolarization. Ajmaline testing was performed in all patients and resulted in J-wave amplification or ST elevation in the inferior leads in a few patients. The latter had a delayed depolarization, whereas no patient with early repolarization had J/ST-wave amplification on ajmaline.

It is noteworthy that in a previous noninvasive mapping study, Zhang et al^62^ did not observe conduction abnormalities in inferolateral J-wave syndrome *during sinus rhythm.* A likely explanation is that the small depolarization fronts that are responsible for the late fractionated electrograms cannot be recognized as such by a mathematical inverse solution from body surface potentials.

**Figure 2:**
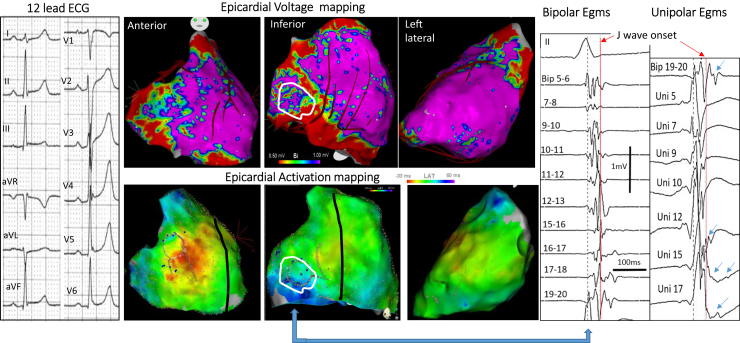
Inferolateral J-wave syndrome due to abnormal depolarization. **Top middle maps** show bipolar electrogram voltage (*purple* indicating voltage >1 mV) with low voltage in inferior right ventricle. **Bottom middle maps** show activation mapping, with *blue* indicating the latest activated regions, here in the inferobasal right ventricle. **Right:** Fragmented electrograms (Egms) preceding and coincident with the J wave (*white contour*) in bipolar and unipolar *(arrows)* mode. **Left:** The 12 lead-ECG in a 19 year- old man who survived VF. ECG = electrocardiogram.

**Figure 3:**
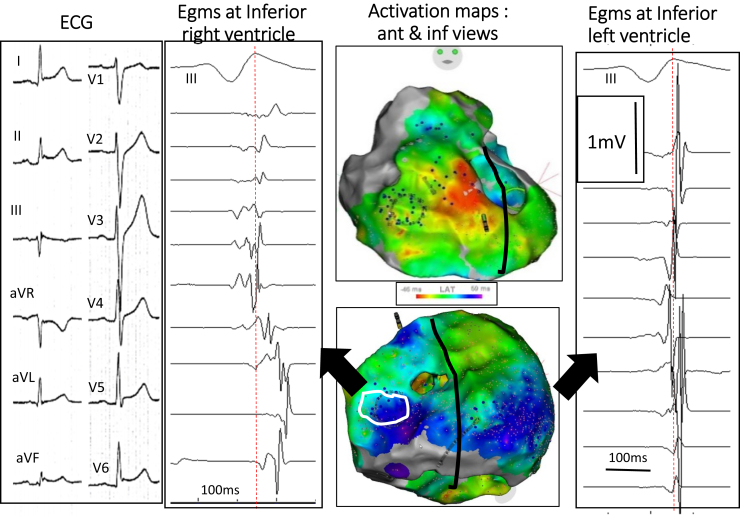
Inferolateral J-wave syndrome due to abnormal depolarization. The maps **(middle)** show activation mapping, with *blue* indicating the latest activated regions, here the inferobasal right and left ventricles. **Right:** Low-voltage fragmented electrograms (Egms) coincident with J wave are only present in the inferior right ventricle (*white contour*) compared with Egms in the inferior left ventricle. **Left:** The 12 lead-ECG in a 31 year- old man who survived VF. ECG = electrocardiogram.

Examples of J waves caused by late depolarization are shown in Figures 2 and 3. The fragmented electrograms occurred timely during the inscription of J waves and were in continuity with the depolarization field. The majority of abnormal electrograms were found on the epicardium (right ventricle and 2 cases in left ventricle), whereas 2 patients had late electrograms recorded endocardially and epicardially. They were recorded predominantly in the inferior right ventricle, at the sites of terminal activation as predicted by a modeling study.^64^ In the patients with BrS and inferolateral J-wave syndromes, the electrograms recorded in the inferior right ventricle were similar to those in the right ventricular outflow tract. The pathogenesis of inferolateral J waves here is dominantly due to abnormal delayed conduction, either limited to the inferior myocardium (right or left, endo- or epicardial) or combined with other locations (eg, anterior right ventricle). The cause responsible for electrogram fractionation (altered myocardial cells or their connections, fibrotic or fatty tissue infiltrations) is undetermined. In addition. not only a heterogeneous delay of activation but also a homogeneous delayed activation may lead to a J-wave.^64^ Finally, there is probably a potential contribution of a repolarization disparity (secondary to the depolarization alterations) to explain a part of J/ST-wave fluctuations or arrhythmogenesis, which requires additional studies.^46^

**Figure 4:**
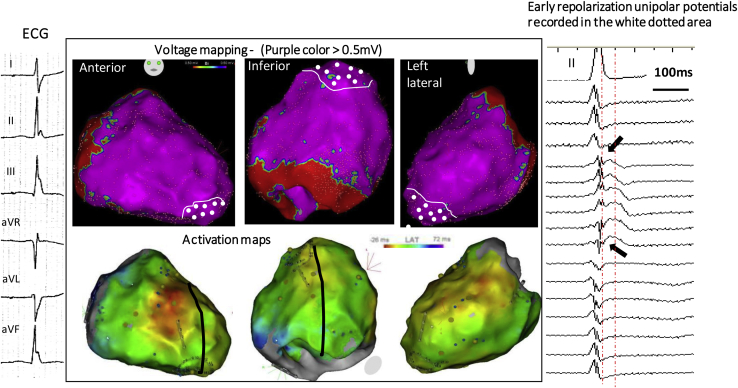
Inferolateral J-wave syndrome due to early repolarization. **Top middle maps** show bipolar electrogram voltage without evidence of low-voltage area. **Bottom middle maps** shows the latest activated regions (*blue*) in the inferobasal and laterobasal right ventricle. **Right:** There are no late depolarization electrograms coincident to the J wave but slow early repolarization potentials (*arrows*), which are present in the apical region (*white-dotted area*). Note that the J wave is small on lead II (right) and underestimates the extent of early repolarization recorded by epicardial mapping. **Left:** A 6 lead- ECG in a 15 year-old girl with recurrent VF. ECG = electrocardiogram.

**Figure 5:**
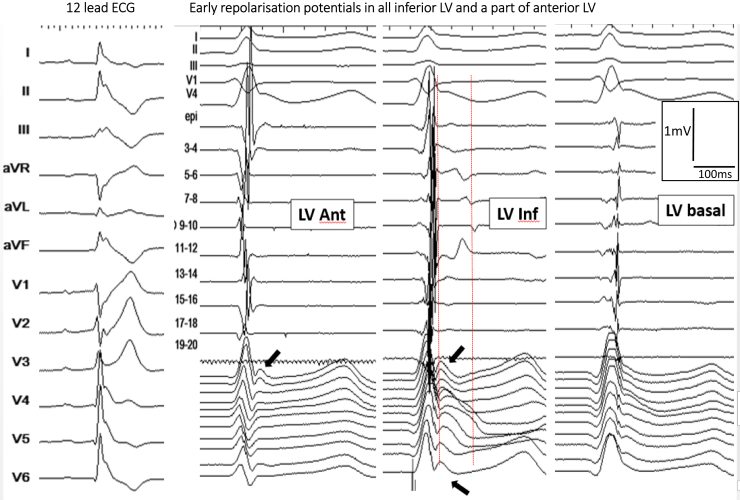
Another case of early repolarization. Twelve-lead ECGs (**left**) show a global J-wave pattern. There are no late depolarization bipolar electrograms coincident with the J wave (between the *red lines*), but early repolarization potentials (*arrows*) are recorded diffusely in the inferior left ventricle (**right**). Ant = anterior; ECG = electrocardiogram; inf = inferior; LV = left ventricle.

Examples of J waves due to (true) early repolarization are shown in Figures 4 and 5. In these patients, we could not find electrograms indicative of delayed depolarization coincident with J waves, but low-frequency (hump) potentials were present at the beginning of the ST segment in unipolar recordings. The spatial location and extent of potentials were epicardially dominant in the inferior septal projection and adjacent left ventricle.^60^, ^61^, ^62^, ^63^ Although other hypotheses cannot be totally excluded,^46^ the J-wave mechanism may represent a lower epicardial voltage across the ventricular wall as shown by Yan, Antzelevitch, and colleagues.^8^, ^14^, ^57^ These authors have emphasized that a short-coupled ectopic beat was “a strong piece of evidence supporting an endo-epicardial myocardial gradient of repolarization leading to phase 2 reentry.”^57^ Such arrhythmogenesis due to abbreviated action potentials (loss of the dome) is technically difficult to demonstrate in clinical conditions. However, we observed that VF is commonly initiated from Purkinje triggers (also with short coupling interval), whereas initiation from myocardial triggers (potentially due to phase 2 reentry) is more particularly observed in patients with multifocal ectopy and widespread early repolarization.^7^

### VF drivers in the 2 forms of inferolateral J-wave syndromes

**Figure 6:**
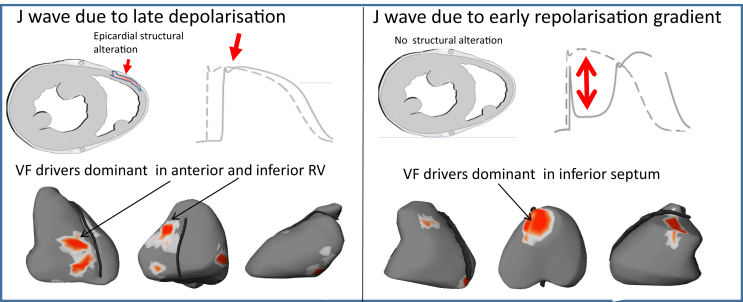
Typical location of ventricular fibrillation (VF) driver regions. The locations of reentries are shown in *red*. They are predominantly located in the right ventricle in late depolarization J waves (**left**) vs the inferior septum in early repolarization (*right*).

VF drivers were mapped using a noninvasive method^59^ and showed dominant drivers in the inferior part of myocardium during the initial stages of VF (Figure 6). VF in patients with J waves indicating delayed depolarization was dominantly associated with drivers in the inferior and anterior right ventricle. In contrast, VF in early repolarization was associated with drivers located in the inferior septum and adjacent regions. Note that this epicardial region overlying the inferior septum may be the breakthrough site of activity originating from the Purkinje posterior fascicle. Importantly, the cycle length of VF (measured at the 10th second after initiation) was significantly shorter in early repolarization than in delayed depolarization (n = 16; 148 ± 5 ms vs 175 ± 4 ms; *P* = .013), consistent with shorter ventricular refractory periods. Supplemental Table 1 summarizes the differences between J waves due to early repolarization vs late depolarization.

### Implications of repolarization vs depolarization origins of inferolateral J wave

The classification of inferolateral J waves in 2 distinct substrates has implications in terms of terminology, pathogeny, genetics, and therapy.

Although the terminology of early repolarization seems adequate in the cases of repolarization abnormality, it is erroneous when the J wave is due to a late depolarization. The term inferolateral J wave may thus be more adequate and generic. Further studies are needed to provide additional phenotypic features that may help distinguish between depolarization and repolarization abnormality. It is likely that a stable J-wave pattern not influenced by long cycle lengths is caused by a depolarization abnormality, potentially exacerbated at short cycle lengths; and vice versa for early repolarization. In a study by Roten et al,^65^ the response of inferior J-wave patterns to isoproterenol varied individually. Inferior J waves were persistent in 35% (rather suggestive of delayed depolarization) and were decreasing or normalizing in 65% of patients (rather suggestive of early repolarization). Baseline QRS width was significantly larger in patients with persistent J waves.^65^

In terms of genetic predisposition, mutations in genes coding for subunits of the I_K-ATP_ channel or cardiac l-type calcium channel have been described, but without validation by functional expression studies in most. Loss-of-function mutations in Na channel genes have also been reported, including a significant proportion of them associated with ST elevation in inferior leads under Na channel blocker. We speculate that a late depolarization J wave will more likely be associated with gene variants in the Na channel, connexins, and structural proteins, whereas mutations in the ion channels carrying I_to_, I_K-ATP_, or I_Ca_ will be more associated with early repolarization.

The immediate benefit of distinguishing J-wave subtypes is its therapeutic potential. In patients with late depolarization J waves, substrate ablation targeting the delayed electrograms is feasible as in BrS and other SHDs. In patients with ER J waves, whether ablation of abnormal repolarizing tissue is applicable and safe is not known, but trigger ablation is an effective option when antiarrhythmic drugs, particularly quinidine, have failed.

### Mechanistic classification of VF associated with apparently normal hearts

The present review shows that similar ECG phenotypes may be caused by fundamentally different substrates. Inferolateral J waves can be the expression of voltage gradients at the initial phase of repolarization (early repolarization) or the expression of delayed depolarized areas. Delayed depolarization is associated with electrogram fractionation indicating local structural alteration, whereas in the early repolarization group, there is likely no structural abnormality, although VF triggers originating from the Purkinje system are often found.

**Figure 7:**
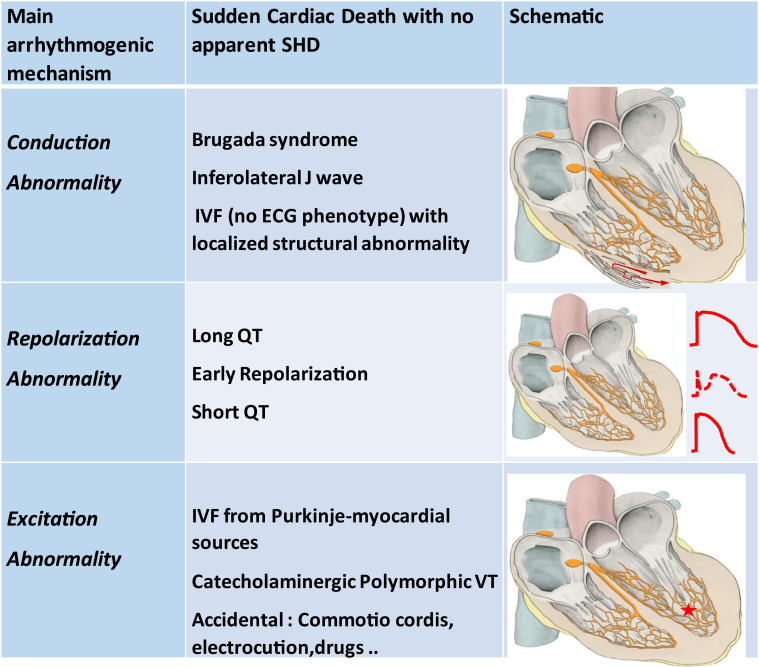
Spectrum of arrhythmogenic diseases leading to sudden cardiac death in apparently normal hearts and proposal of a mechanistic classification based on the primary pathogenesis. IVF = idiopathic ventricular fibrillation; VT = ventricular tachycardia.

Similar findings have been reported recently in idiopathic VF, using high-density electrogram characterization.^59^ The study involved 24 patients with no ECG phenotype. J waves and long or short QT syndrome were excluded, and imaging and ajmaline testing were strictly negative. Localized areas of abnormal depolarization were identified in 62% of the patients. Most abnormal areas were epicardial, affecting dominantly the right ventricle, and of limited size (5% of the total surface). In the remaining subset of patients with idiopathic VF devoid of myocardial alteration (38% of patients), Purkinje triggers were evidenced and seemed to be the dominant mechanism. Therefore, the spectrum of arrhythmogenic diseases leading to SCD in apparently normal hearts, including the J-wave syndromes, seems to comprise an important emerging subgroup in which the dominant substrates are localized depolarization abnormalities that may or may not have an ECG expression. A simplified mechanistic classification based on the primary pathogenesis is proposed in Figure 7.

## Conclusion

Inferolateral J waves are subtle ECG phenotypes that may be responsible for SCD in patients with no apparent SHD. Their occurrence at the QRST junction can be the expression of distinct substrates, early repolarization or delayed activation abnormality, or mixed forms. Distinguishing between these substrates could significantly improve genetic interpretation, risk stratification, and the therapeutic approach. Further studies are needed for quantitative assessment of the recorded signals and determination of the pathogenesis of inferolateral J waves.
