# Supplementary material for: Depolarization versus repolarization abnormality underlying inferolateral J-wave syndromes: New concepts in sudden cardiac death with apparently normal hearts
Source: Heart Rhythm. 2019 May;16(5):781–90. doi: 10.1016/j.hrthm.2018.10.040 (PMC6486498; doi:10.1016/j.hrthm.2018.10.040)
Supplement: Supplemental Table 1 [file mmc1.docx]

**TABLE 1. Differences between J waves due to early repolarization versus late depolarization**

|  | Early repolarization | Late depolarization |
| --- | --- | --- |
|  |  |  |
| Mechanisms | Repolarization gradient at the early phase (likely) | Depolarization abnormality- at the terminal QRS complex |
| Structural heart disease | Absent | Present |
| Egm abnormality coincident with J wave | No sharp fragmented egms  Unipolar low frequency potential (hump or ST elevation) at early repolarization, | Fragmented egms in Epicardial RV or LV  may be located endocardially  Associated slow potential hump is possible |
| Location of Egm abnormality | Inferior walls of RV/Septum/ LV – plus adjacent regions in widespread J wave | Sites of latest activation at infero basal RV or LV  Mostly at epicardial side |
| J wave morphology | Expected to be variable  A hump-shaped (Osborn) J wave is likely specific | Expected to be more stable |
| Response to cycle length heart rate | Increase after longer R-R cycles (Bradycardia- and after pauses)  Decrease at shorter cycles (exercise testing) | Unchanged or increased at shorter R-R cycles  May decrease at longer cycles |
| VF trigger | Purkinje dominant (likely) | Myocardial dominant? |
| Main VF drivers | Inferior Septum and adjacent RV/LV regions  Purkinje - papillary muscles? | Inferior and anterior walls of RV |
| VF CL at 10^th^ sec | Short | Longer |
| Drug testing with Na channel blocker | J wave reduced | J wave unchanged  or amplified with ST elevation |
| Quinidine effect | Effective | Likely lesser effect |
|  |  |  |
|  |  |  |
